# Experimental modulation of Interleukin 1 shows its key role in chronic kidney disease progression and anemia

**DOI:** 10.1038/s41598-021-85778-2

**Published:** 2021-03-18

**Authors:** Inbar Bandach, Yael Segev, Daniel Landau

**Affiliations:** 1 Shraga Segal Department of Microbiology and Immunology, Faculty of Health Sciences, Ben Gurion University of the Negev, Beer Sheva, Israel; 2 Institute of Nephrology, Schneider Children’s Medical Center of Israel, 14 Kaplan Street, 4920235 Petach Tikva, Israel; 3 Sackler School of Medicine, Tel Aviv University, Tel Aviv, Israel

**Keywords:** Chronic kidney disease, Anaemia, Chronic inflammation, Inflammation, Immunology, Nephrology

## Abstract

Inflammation in chronic kidney disease (CKD) is mostly due to activation of the innate immune system, in which Interleukin-1 (IL-1) is a key player. Anemia of CKD may also be due to erythropoietin (EPO) resistance, clinically associated with inflammation. IL-1 receptor antagonist knockout (RaKO) mice show arthritis and excessive inflammation. Inhibition of IL-1 was shown to be beneficial in many inflammatory conditions, but its role in CKD and anemia is unknown. Here, we report that enhanced inflammation in RaKO mice with CKD provoked both higher degrees of renal insufficiency and anemia in comparison to wild-type CKD, in association with a downregulation of renal hypoxia inducible factor-2 (HIF2) as well as decreased bone marrow EPO-receptor (EPOR) and transferrin receptor (TFR). In contrast, administration of P2D7KK, an anti-IL1b monoclonal antibody, to CKD mice results in a lower grade of systemic inflammation, better renal function and blunted anemia. The latter was associated with upregulation of renal HIF-2α, bone marrow EPO-R and TFR. Altogether, this supports the key role of inflammation, and IL-1 particularly, in CKD progression and anemia. Novel treatments to reduce inflammation through this and other pathways, may improve renal function, attenuate the anemic state or increase the response to exogenous EPO.

## Introduction

Anemia is a common feature of progressive chronic kidney disease (CKD), associated with its other complications^1^. It was originally thought to be due to absolute or impaired renal erythropoietin (EPO) synthesis and disordered iron homeostasis caused by blood losses and inflammation-related disrupted iron metabolism, mediated by hepcidin^2,3^. Current treatment of anemia in CKD includes erythropoiesis-stimulating agents (ESA) and/or oral or intravenous (IV) iron supplementation. However, clinical trials demonstrated increased morbidity and mortality related to aggressive ESA treatment^4^. In addition, 10%-20% of CKD patients are resistant to ESA^5^, which has been clinically associated with the inflammatory state^6–8^, where elevated levels of cytokines related to the innate immune system prevail^9^. IL-1 is a key upstream pro-inflammatory cytokine that plays essential roles in acute and chronic inflammation, host defense and acute phase responses, enhancing inflammatory cell infiltration, and augmenting adhesion molecule expression^10–12^. IL-1 induces cytokines secretion such as IL-6, leading to enhanced STAT3 transcriptional activity^13,14^. IL-1α, IL-1β and IL-1 receptor antagonist (Ra) are members of the IL-1 family^11,15^. Binding of IL-1 Ra to IL-1R has no IL-1-like activity^16,17^. IL-1Ra knockout (RaKO) mice spontaneously develop a rheumatoid arthritis-like chronic inflammatory state^18^. Blocking IL-1β with canakinumab has been approved for use in relapsing and chronic inflammatory diseases^19^. P2D7KK, a novel monoclonal antibody, has a strong affinity for both human and murine IL-1β, resulting in its robust neutralization^20^. P2D7KK treatment abolished collagen antibody-induced arthritic symptoms, reduced monosodium urate-induced peritonitis and prevented lymphocyte infiltration in a collagen antibody induced arthritis mouse model^20^. Human studies have shown efficacy for canakinumab treatment in the reduction of adverse cardiovascular outcomes in adults with atherosclerosis, especially those with elevated C-reactive protein^21^, including in patients with underlying mild CKD^22^. However, no data exist on the effects of IL-1 inhibition on renal damage extent or anemia control in CKD.

The purpose of this study was to characterize the renal phenotype and anemic state in CKD, modulating inflammation using an inflammation-prone animal model (RaKO mice) in comparison to anti- IL-1β antibody treatment.

## Results

See supplementary Fig. S1 for a summary of the experiments and their main results.

### Worsening anemia in IL-1 receptor antagonist knock-out mice with CKD

IL-1 RaKO mice spontaneously develop chronic inflammatory arthritis^18^, which was also seen in our experiment. This arthritis was aggravated in the RaKO-CKD group: swelling and redness were observed in the ankle joints, which were also filled with pus-like material (Supplementary Fig. S2).

**Figure 1:**
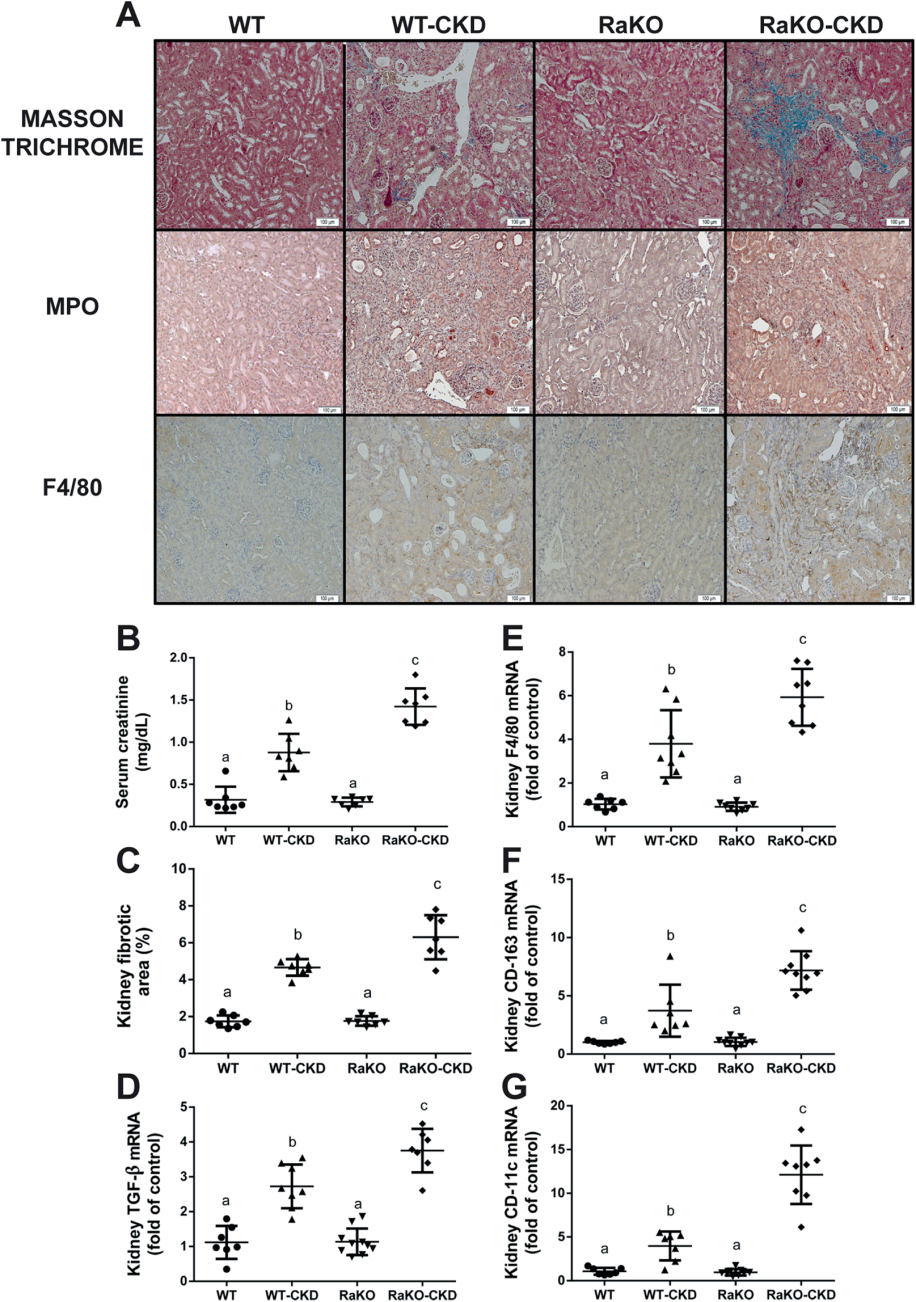
Renal phenotype and fibrosis of CKD mice with uncontrolled IL1 expression. Experimental groups include: wild type mice on a regular (WT) or adenine diet (WT-CKD), IL1-R antagonist knockout mice on regular diet (RaKO) or adenine (RaKO-CKD). Representative kidney section **(A).** Upper row: Masson Trichrome staining; Middle row: immunohistochemical (IHC) staining for neutrophils (Myeloperoxidase—MPO); Lower row: IHC staining for macrophages (F4/80). Bar = 100 μm. Serum creatinine (mg/dL) **(B)**. Kidney fibrotic area percentage as determined by the extent of Masson Trichome staining, analyzed with the *ImageJ* software **(C)**. Kidney TGF-β mRNA levels **(D)**. Kidney F4/80 **(E),** CD-163 **(F)** and CD-11c **(G)** mRNA levels. n = 6–10 per group. Different letters above bars indicate a significant difference between groups, similar letters indicate no significant difference.

CKD was induced by the adenine diet, which causes crystal deposition (Supplementary Fig. S3) and inflammation around them in tubulointerstitial areas. The crystals are washed out by the tissue fixation, leaving irregular cavities in the tubulointerstitium. Higher levels of tubulointerstitial fibrosis were seen in RaKO-CKD Vs. WT-CKD (Fig. 1A,C). The Inflammatory infiltration was composed mainly of macrophages (immunostained with F4/80) and not of polymorphonuclear cells (immunostained with Myeloperoxidase, MPO) (Fig. 1A). Serum creatinine and kidney TGF-β mRNA levels were more elevated in RaKO-CKD Vs. WT-CKD (Fig. 1B,D). Kidney macrophage markers (F4/80, CD-163 and CD11c) mRNA levels were increased in RaKO-CKD Vs. WT-CKD (Fig. 1E–G).

**Figure 2:**
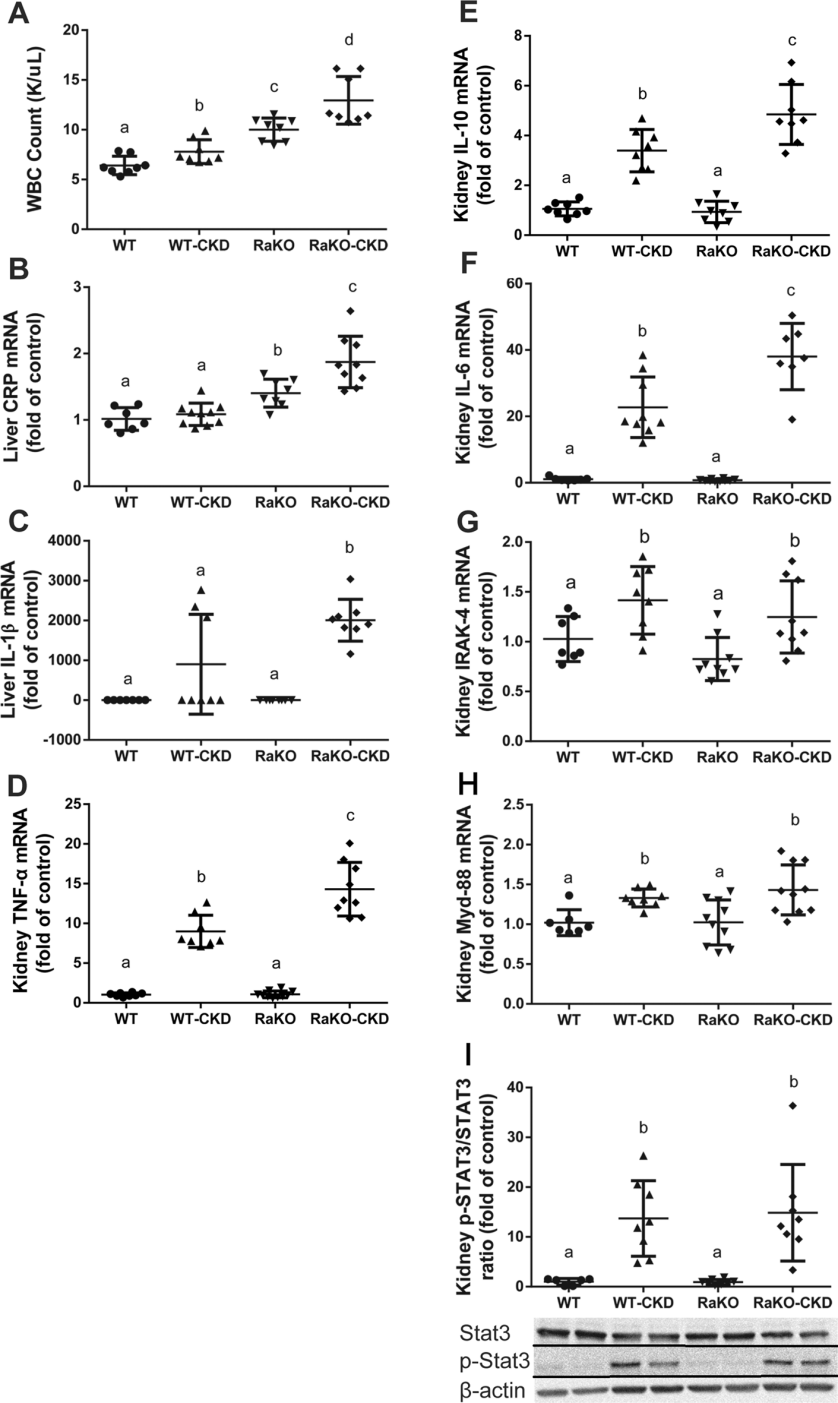
Systemic inflammation of CKD mice with uncontrolled IL1 expression. Experimental groups include: wild type mice on a regular (WT) or adenine diet (WT-CKD), IL1-R antagonist knockout mice on regular diet (RaKO) or adenine (RaKO-CKD). White blood cell count (K/uL) **(A).** Liver C-reactive protein (CRP) **(B)** and Interleukin-1 β (IL-1β) **(C)** mRNA levels. Kidney TNF-α **(D)**, Interleukin-10 (IL10) **(E),** Interleukin-6 (IL6) **(F)**, IRAK-4 **(G)** and MYD-88 **(H)** mRNA levels. Kidney p-STAT3/STAT3 protein ratio (corrected for β-actin). The lower panel shows a representative gel **(I)**. n = 7–10 per group. Different letters above bars indicate a significant difference between groups, similar letters indicate no significant difference.

Peripheral blood leukocyte (WBC) count was increased in WT-CKD Vs. WT and in both RaKO groups, especially in RaKO-CKD (Fig. 2A). Liver CRP mRNA levels were increased in both RaKO groups Vs. WT groups, especially in RaKO-CKD (Fig. 2B).

Kidney TNF-α, IL-10 and IL-6 mRNA levels were markedly increased in both CKD groups, especially in RaKO-CKD (Fig. 2D–F). Also, kidney IRAK-4 and Myd-88 mRNA levels and kidney phospho-STAT3 protein levels were increased significantly in CKD groups compared to controls (Fig. 2G–I).

**Figure 3:**
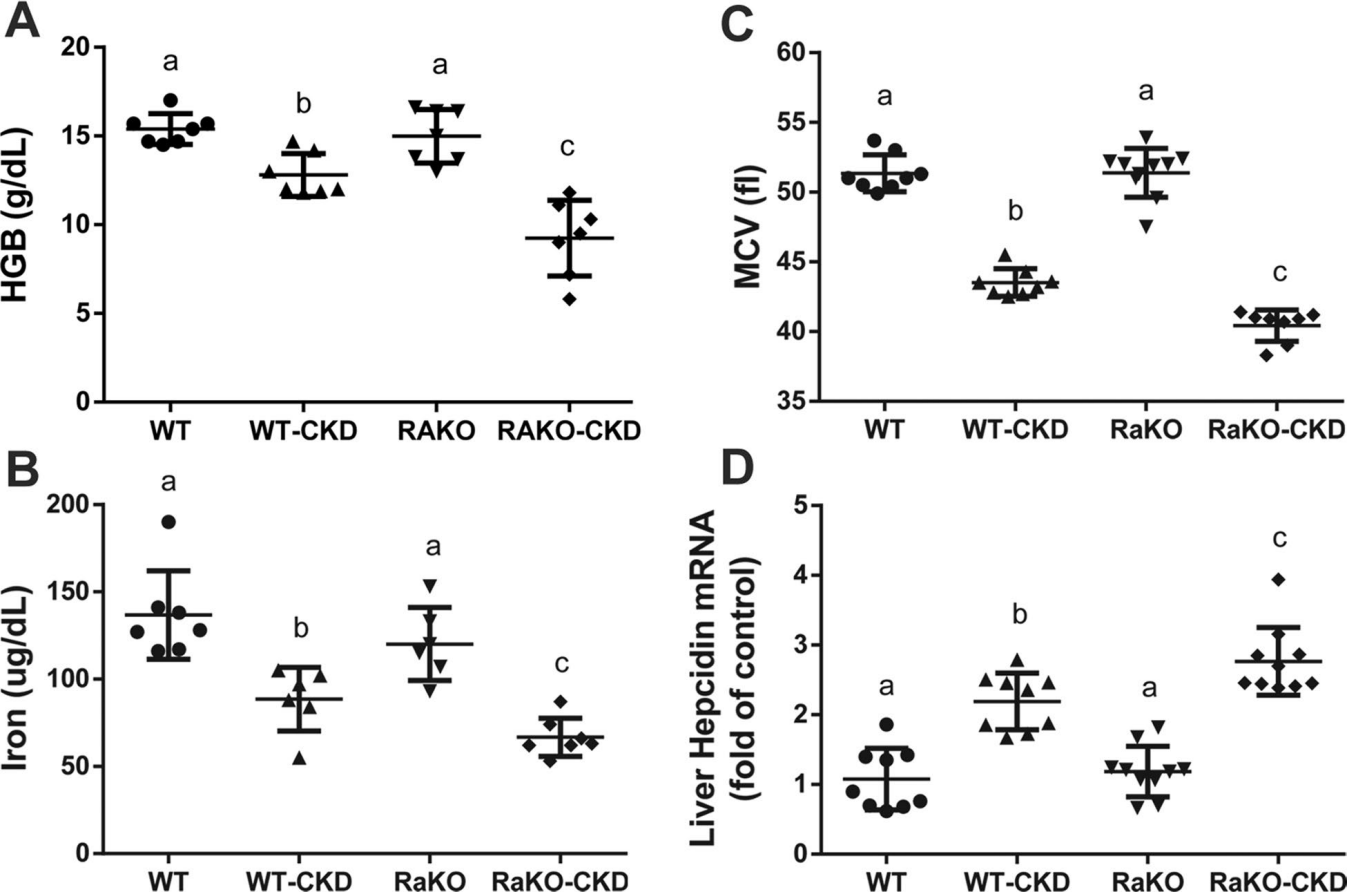
Anemia parameters of CKD mice with uncontrolled IL1 expression. Experimental groups include: wild type mice on a regular (WT) or adenine diet (WT-CKD), IL1-R antagonist knockout mice on regular diet (RaKO) or adenine (RaKO-CKD). Hemoglobin (g/dL) **(A)**. Serum Iron levels (ug/dL)** (B).** Mean corpuscular volume (fl) **(C)**. Liver hepcidin mRNA levels **(D)**. n = 6–10 per group. Different letters above bars indicate a significant difference between groups, similar letters indicate no significant difference.

**Figure 4:**
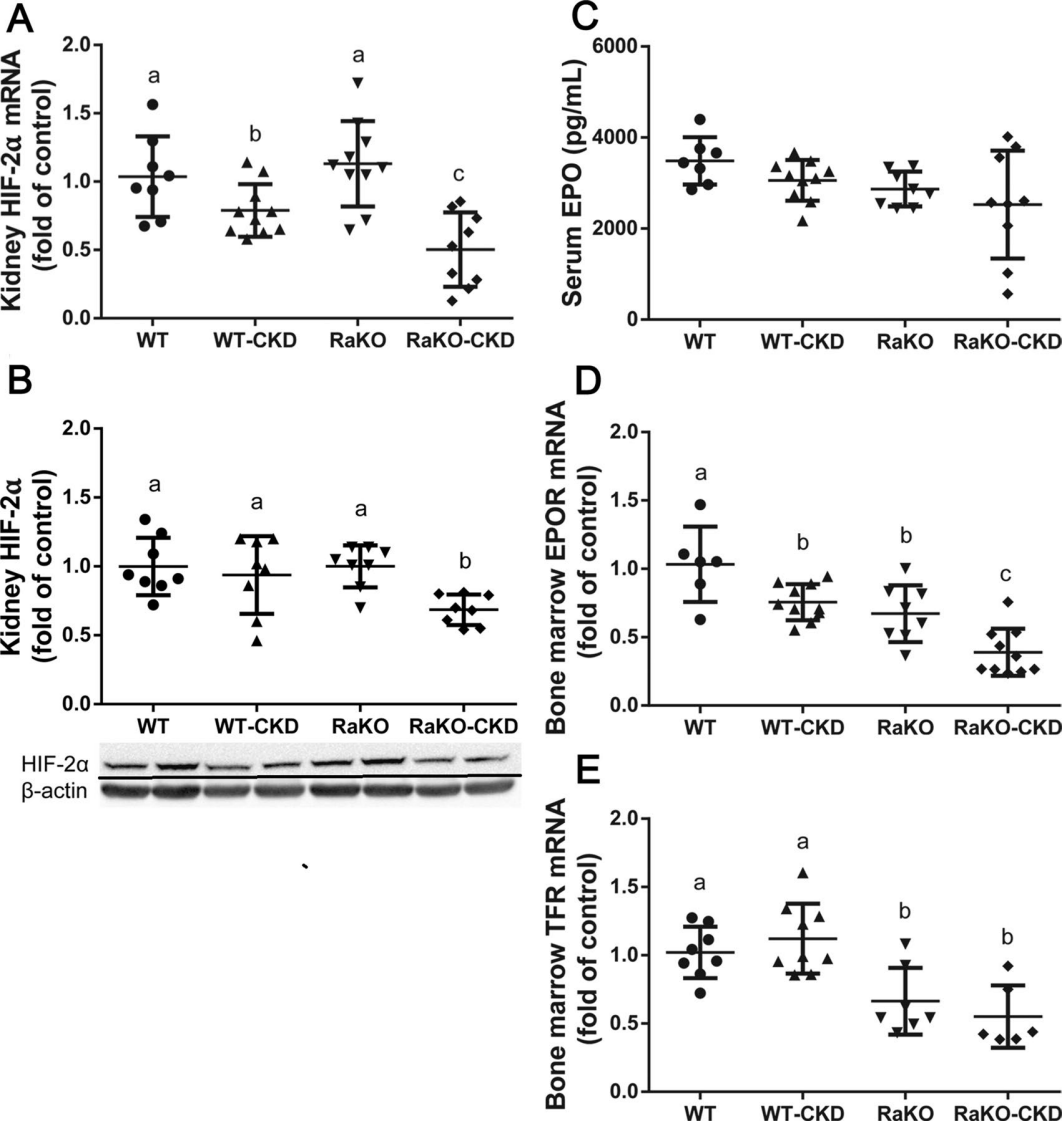
HIF2-EPO-EPO-R response of CKD mice with uncontrolled IL1 expression. Experimental groups include: wild type mice on a regular (WT) or adenine diet (WT-CKD), IL1-R antagonist knockout mice on regular diet (RaKO) or adenine (RaKO-CKD). Kidney hypoxia-inducible factor 2α (HIF2a) mRNA levels **(A)** and protein levels (normalized for β-actin). The lower panel shows a representative gel **(B)**. Serum erythropoietin (EPO) levels (pg/mL) **(C)**. Bone marrow EPO receptor (EPOR) **(D)** and transferrin receptor (TFR) **(E)** mRNA levels. n = 6–10 per group. Different letters above bars indicate a significant difference between groups, similar letters indicate no significant difference.

Hemoglobin (Hgb) levels were decreased in CKD groups, especially in RaKO-CKD (Fig. 3A). Serum iron and mean corpuscular volume (MCV) levels were also significantly reduced in both CKD groups and were even lower in RaKO-CKD Vs. WT-CKD (Fig. 3B–C). Liver hepcidin mRNA levels were increased in both CKD groups, especially in RaKO-CKD (Fig. 3D). Contrary to an appropriate response to anemia and tissue hypoxia^31^, renal HIF-2α mRNA and protein levels were significantly decreased in RaKO-CKD group (Fig. 4A,B). Serum EPO levels were unchanged in the CKD groups (Fig. 4C). Bone marrow EPO-R mRNA levels were significantly decreased in WT-CKD, especially in RaKO-CKD (Fig. 4D). Bone marrow transferrin receptor (TFR) mRNA levels were also significantly reduced in RaKO and RaKO-CKD in comparison to the WT group (Fig. 4E).

### Effects of anti-IL-1β antibody on CKD

**Figure 5:**
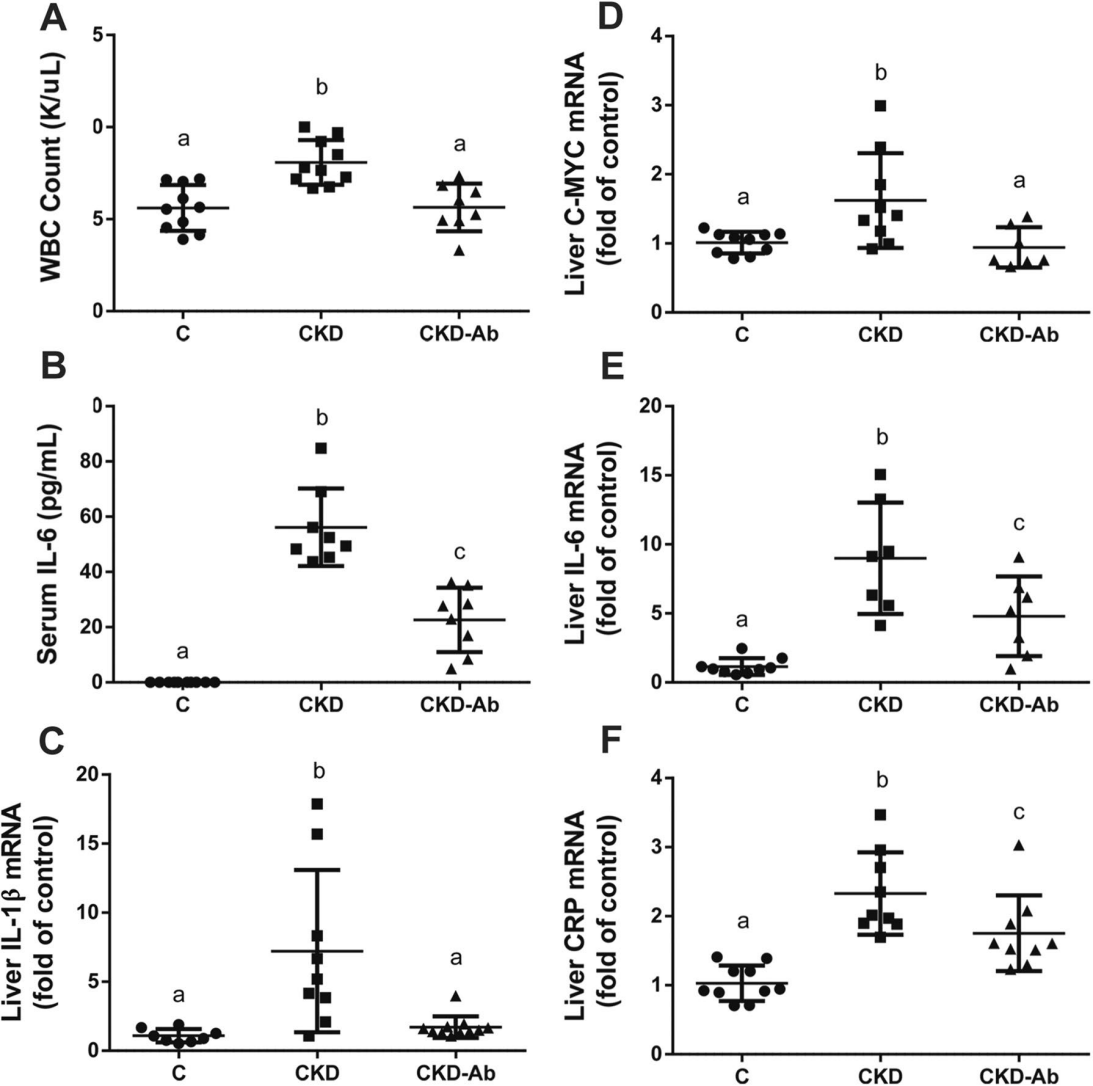
Response of CKD mice to IL1 inhibition. Experimental groups included mice on regular (C) or adenine diet, injected with saline (CKD) or an anti-IL1 monoclonal antibody (CKD-Ab). White blood cell count (K/$\upmu$L) **(A)**. Serum interleukin-6 levels (pg/mL) **(B)**. Liver Interleukin-1β (IL-1β) **(C)**, c-MYC **(D)**, interleukin-6 **(E)** and C reactive protein (CRP) **(F)** mRNA levels. n = 7–10 per group. Different letters above bars indicate a significant difference between groups, similar letters indicate no significant difference.

C57BL/6 mice were divided into 3 groups: C, CKD and CKD-Ab, the latter injected with anti IL-1 monoclonal antibody (P2D7KK). Weight loss was seen in both CKD groups Vs. C, but it was significantly blunted in CKD-Ab Vs. CKD (3.2 ± 0.2 Vs -4.4 ± 0.4 Vs -3.1 ± 0.1 g, p < 0.0001; Supplementary Figure S4). Peripheral WBC count levels were increased in CKD in comparison to C but were normalized in the CKD-Ab group (Fig. 5A). Furthermore, serum IL-6, liver IL-1β, c-MYC, IL-6 and CRP mRNA levels were increased in CKD group but were decreased in CKD-Ab Vs. CKD (Fig. 5B–F).

**Figure 6:**
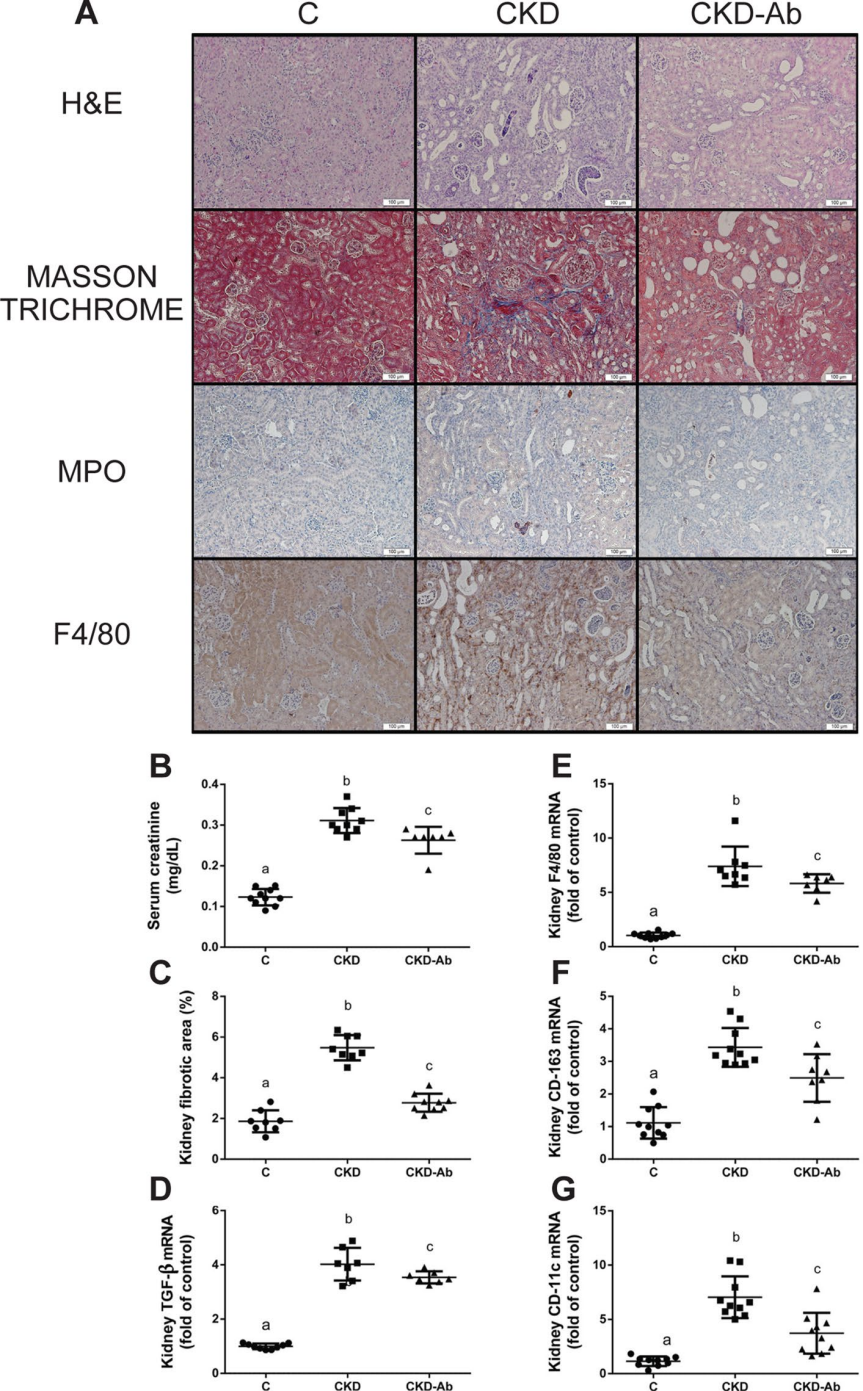
Renal changes in CKD mice following IL1 inhibition. Experimental groups included mice on regular (C) or adenine diet, injected with saline (CKD) or an anti-IL1 monoclonal antibody (CKD-Ab). Representative kidney sections **(A)**, Upper 2 rows: kidney sections stained with hematoxylin & eosin and Masson Trichrome. Lower 2 rows: immunohistochemical staining for neutrophils (Myeloperoxidase—MPO) and macrophages (F4/80). Bar = 100 μm. Serum creatinine (mg/dL) **(B)**. Kidney fibrotic area percentage as determined by the extent of Masson Trichome staining, analyzed with the *ImageJ* software **(C)**. Kidney TGF-β **(D)**, F4/80** (E),** CD-163 **(F)** and CD-11c **(G)** mRNA levels. n = 6–10 animals per group. Different letters above bars indicate a significant difference between groups, similar letters indicate no significant difference.

The elevation of serum creatinine was significantly suppressed in CKD-Ab Vs. CKD (Fig. 6B). In addition, similar to the previous experiment (Fig. 1), immunostainable kidney F4/80, as well as kidney macrophage markers: F4/80, CD-163 and CD-11c mRNA levels, showed increased macrophage recruitment in the CKD group in comparison to C, which was significantly lower in CKD-Ab group (Fig. 6A,E–G). In contrast, neutrophils (MPO positive) infiltration was minimally observed in CKD and CKD-Ab groups (Fig. 6A). Moreover, a decrease in interstitial fibrosis was seen in CKD-Ab in comparison to CKD (Fig. 6A,C–D).

**Figure 7:**
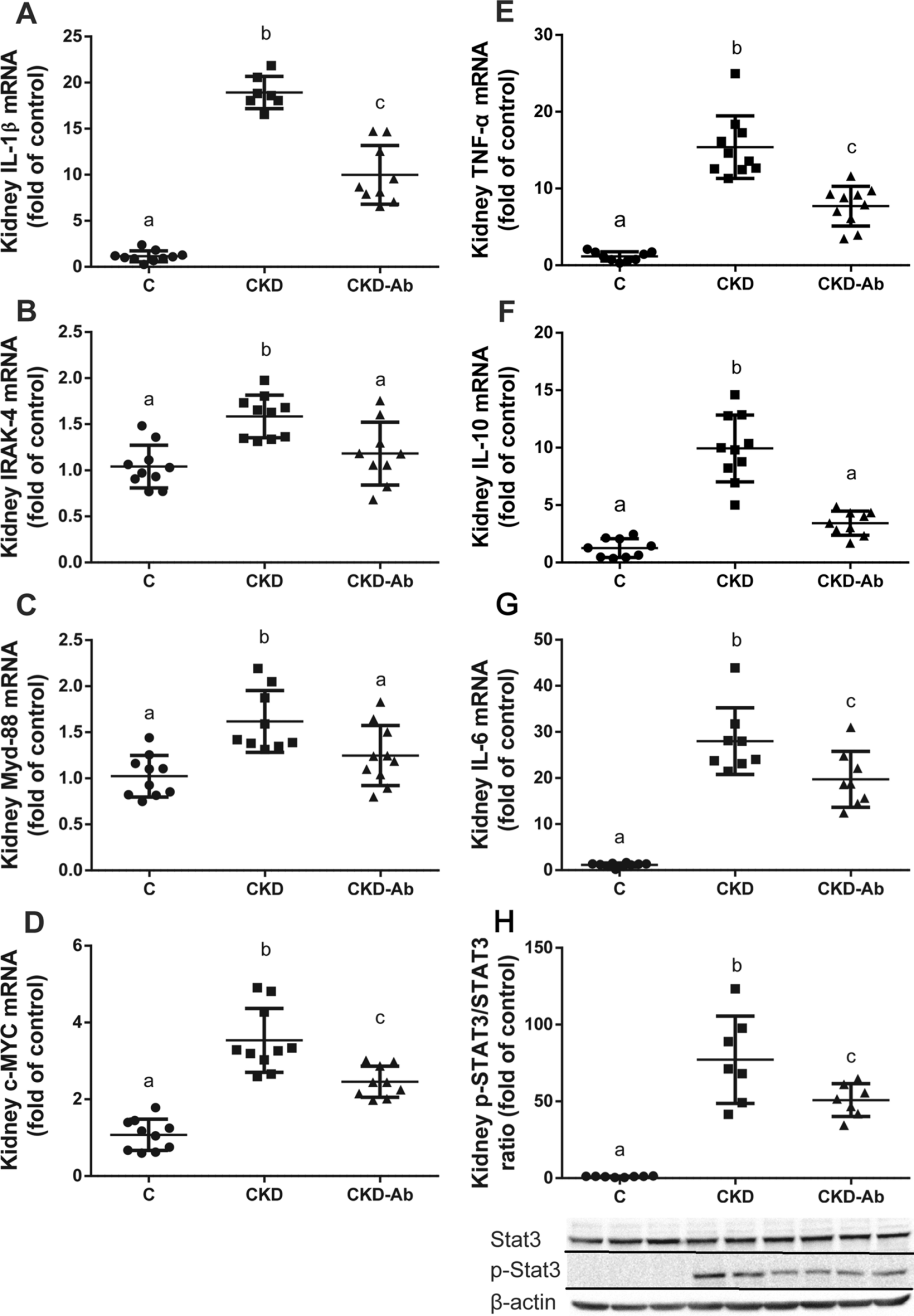
Renal inflammatory response of CKD mice to IL1 inhibition. Kidney inflammation in mice on regular (C) or adenine diet, injected with saline (CKD) or anti-IL1 monoclonal antibody (CKD-Ab). Kidney Interleukin-1β **(A)**, IRAK-4 **(B)**, MYD-88 **(C)**, c-MYC **(D)**, Kidney TNF-α **(E)**, Interleukin-10 (IL10) **(F),** and interleukin-6 (IL6) **(G)** mRNA levels. Kidney p-STAT3/STAT3 protein ratio (normalized for β-actin). The lower panel shows a representative gel **(H)**. n = 7–10 animals per group. Different letters above bars indicate a significant difference between groups, similar letters indicate no significant difference.

Kidney inflammation (TNF-α, IL-10, IL-6 and its downstream molecule p-STAT3, IL-1β and its downstream molecules IRAK-4, MYD-88 and C-MYC), which were significantly elevated in CKD Vs. C, were significantly decreased in CKD-Ab in comparison to CKD (Fig. 7).

**Figure 8:**
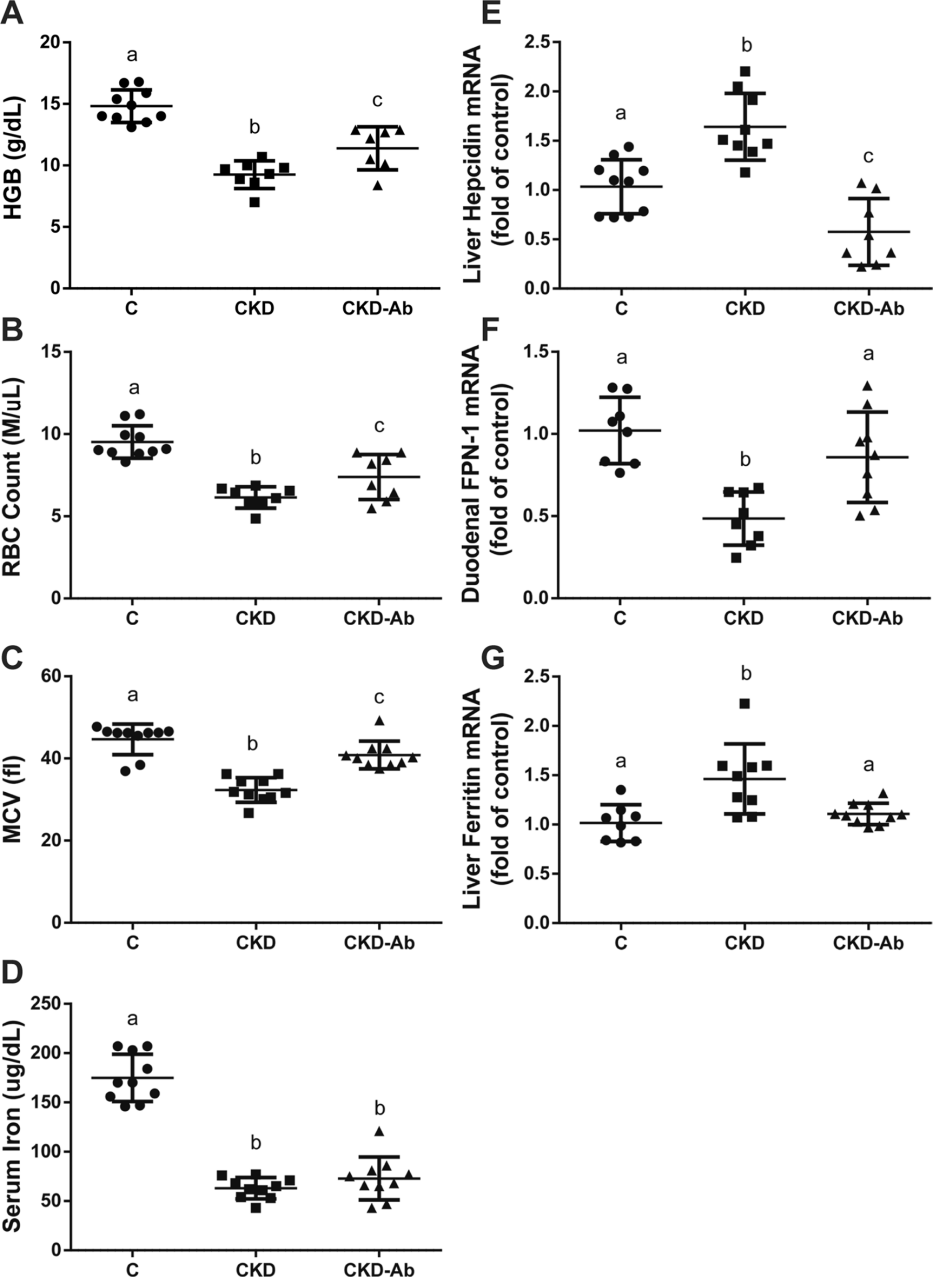
Anemia response of CKD mice to IL1 inhibition. Anemia and iron parameters in mice on regular (C) or adenine diet, injected with saline (CKD) or an anti-IL1 monoclonal antibody (CKD-Ab). Hemoglobin (g/dL) **(A)**. Red blood cell count (M/μL) **(B)**. Mean corpuscular volume (fl) **(C)**. Serum Iron levels (ug/dL) **(D)**. Liver hepcidin mRNA levels **(E)**. Duodenal ferroportin-1 mRNA levels **(F)**. Liver ferritin mRNA levels **(G)**. n = 7–10 animals per group. Different letters above bars indicate a significant difference between groups, similar letters indicate no significant difference.

**Figure 9:**
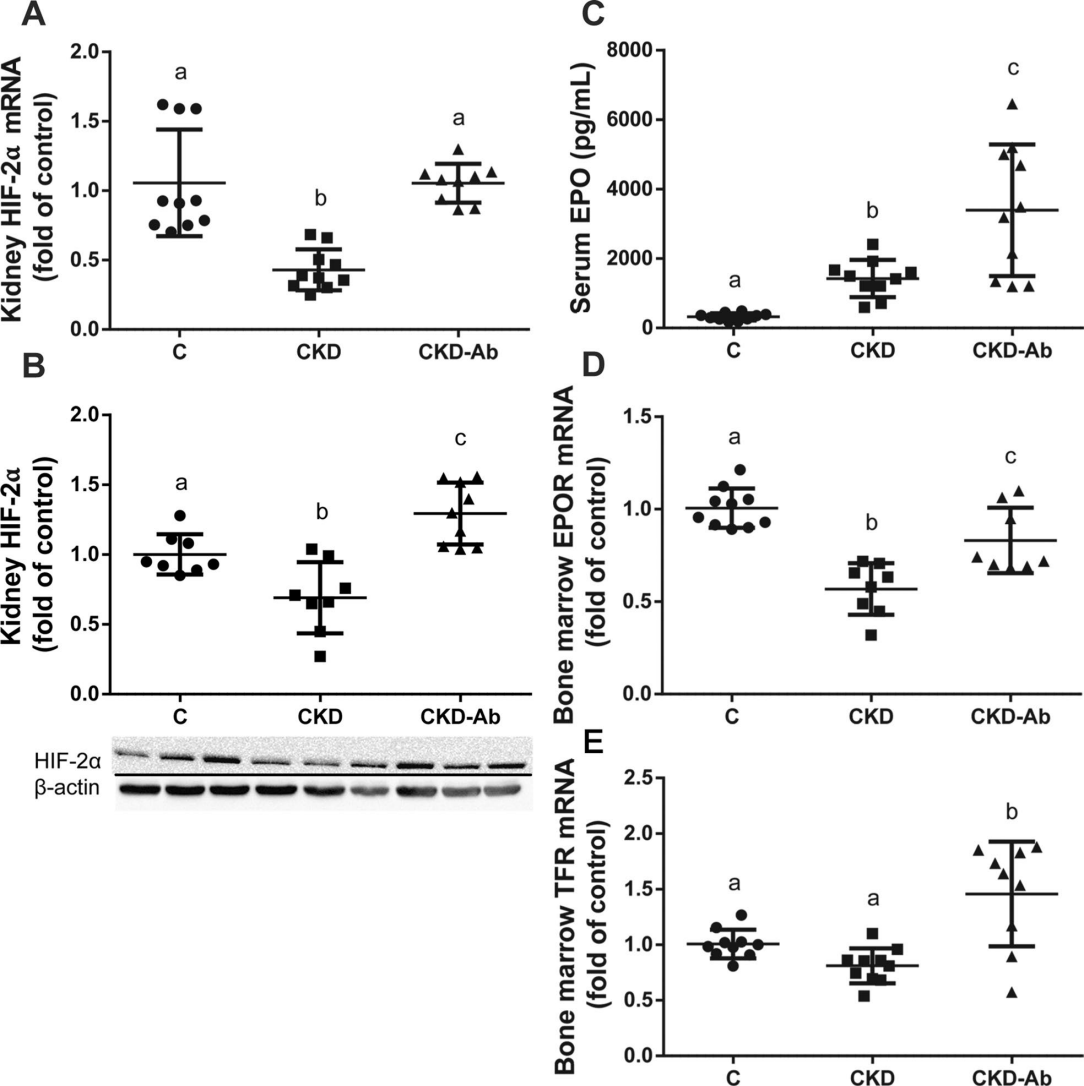
HIF2-EPO-EPO-R response of CKD mice to IL1 inhibition. Experimental groups included mice on regular (C) or adenine diet, injected with saline (CKD) or an anti-IL1 monoclonal antibody (CKD-Ab). Kidney hypoxia-inducible factor 2α (HIF-2α) mRNA levels **(A)** and protein levels (normalized for β-actin). The lower panel shows a representative gel **(B)**. Serum erythropoietin (EPO) levels (pg/mL) **(C)**. Bone marrow EPO receptor (EPOR) **(D)** and transferrin receptor (TFR) **(E)** mRNA levels. n = 7–10 animals per group. Different letters above bars indicate a significant difference between groups, similar letters indicate no significant difference.

Hemoglobin levels, red blood cell (RBC) count and MCV levels were decreased in CKD animals but improved in CKD-Ab Vs. CKD (Fig. 8A–C). Serum iron levels remained decreased in both CKD groups (Fig. 8D). Liver hepcidin mRNA, which was increased in CKD Vs. C, decreased in CKD-Ab Vs. CKD (Fig. 8E). In addition, duodenal ferroportin (FPN-1) mRNA levels, which play a role as iron transporter and is inversely correlated with hepcidin, increased in CKD-Ab Vs. CKD (Fig. 8F). Liver ferritin mRNA levels decreased in CKD-Ab Vs. CKD (Fig. 8G). Renal HIF-2α mRNA and protein levels and bone marrow EPO-R mRNA levels were significantly reduced in both CKD groups but increased in CKD-Ab Vs. CKD (Fig. 9A,B,D). Bone marrow TFR mRNA levels were significantly increased in CKD-Ab Vs CKD (Fig. 9E).

## Discussion

We describe here that anemia and renal damage extent can be modulated (both worsened as well as improved) by the degree of IL-1 expression in a mouse model of CKD. In the first part of this study, we show pleiotropic effects on the general inflammatory state, kidney function and fibrosis, anemia, iron metabolism and HIF-EPO-EPO-R axis in uremic IL-1Ra knockout (RaKO) mice, which spontaneously develop a chronic inflammatory polyarthropathy due to an uncontrolled IL-1 action^18^. Biomarkers of inflammation (IL-1β, IL-1Ra, IL-6, and CRP) have been clinically reported to be inversely associated with measures of kidney function^9^. IL-6 and IL-1 stimulate fibrosis in kidney tissue^23,24^. In our experiments, CKD-RaKO mice show elevated circulating leukocytes, liver CRP, liver IL-1β and kidney TNF-α, IL-10 and IL-6 in comparison to WT-CKD (Fig. 2), as well as worse renal function and fibrosis, tubular atrophy and dilatation, tissue damage and macrophage infiltration (Fig. 1). Monocyte/macrophage cells have been reported to play an active and essential role in tubulointerstitial fibrosis^25^. Tubulointerstitial damage severity is known to best correlate with renal function's decline^26^.

We have previously reported that anemia in juvenile CKD rats is associated with inappropriate responses in the HIF-EPO-EPO-R axis^27^: the typical response to anemia induced hypoxia (increased HIF2 leading to increased renal EPO, which controls EPO-R and TFR expression) was shown to be deranged in CKD. The current study now shows an accentuated anemia when inflammation is further increased in the RaKO-CKD group in comparison to WT-CKD (Fig. 3A). The effects of CKD on iron absorption and mobilization are well known, mainly through the upregulation of liver hepcidin, an essential player in the development of anemia of CKD^3^ as well as other chronic inflammatory conditions^28^. It regulates iron homeostasis by both inhibiting intestinal iron absorption and iron release from macrophages^29^. IL-6 and IL-1 have been shown to induce hepcidin transcription^30,31^. Anemic phenotype is ameliorated in hepcidin knockout juvenile CKD mice^32^. In our experiments, liver hepcidin levels were elevated in the inflamed and uremic RaKO-CKD mice (Fig. 3D), which may have led to the lower levels of serum iron and MCV in comparison to WT-CKD (Fig. 3B,C). Interestingly, RaKO animals showed unchanged hemoglobin, hepcidin and iron levels (Fig. 3) in spite of increased systemic inflammation (Fig. 2A,B) in comparison to WT animals. No distinct renal phenotype was seen either. These mice usually exhibit some growth retardation and arthritis^18,33^. Here, the addition of a uremic state seems to have worsened arthritis (see Supplementary Figure S2) as well as systemic and local renal inflammation. Similar organ-specific damage driven by the IL-1 pathway has been previously shown in a mouse model of cystic fibrosis^34^.

Despite the decreased levels of hemoglobin in both CKD groups (but further accentuated in RaKO-CKD, Fig. 3A), serum EPO levels were unchanged (Fig. 4C), suggesting an inappropriate HIF2-EPO pathway signal. Indeed, renal HIF-2α protein and mRNA levels were decreased in the RaKO-CKD group (Fig. 4A).

Although EPO deficiency is likely the main determinant of anemia in patients with advanced CKD, Mercadal et al. have shown that anemia in CKD patients with eGFR > 30 ml/min should be explained by other factors^35^. In addition Krause et al. have shown that the higher prevalence of anemia in post- kidney transplantation patients could not be explained by an iron deficiency or reduced EPO production^36^. Therefore, anemia of CKD seems to be multifactorial rather than just EPO deficiency, and this could be the reason for the variability in serum EPO levels in our uremic groups.

Chiang et al*.* provided evidence that links uremic toxins to the suppression of EPO gene transcription in hypoxic HepG2 cells in a HIF-dependent manner^37^. Suoma et al*.* have reported that renal EPO producing cells possess cellular plasticity, which is modulated by inflammatory molecules^38,39^. In our experiments, the decrease in bone marrow EPO-R and TFR in RaKO-CKD could lead to a reduction in the activity of EPO as well as iron bone marrow availability (Fig. 4D-E). IL-1, IL-6 and interferon-gamma affect EPO/EPOR axis via downregulation of EPOR expression on erythroid precursor cells *in-vitro*^40,41^. IL-1β suppresses EPO gene expression in isolated perfused rat kidneys and human hepatoma cell cultures^42^. Also, IL-1 inhibited human erythroid colony-forming units by the mediation of gamma interferon^43^. Thus, accelerating inflammation through IL-1 in CKD mice could lead to disrupted iron metabolism and downregulation of renal HIF-2α and EPO as well as iron availability, with a secondary decrease in bone marrow EPO-R and TFR, which altogether further exacerbate anemic tendency.

To further show the role of IL-1 in CKD related renal damage extent and anemic state, we show the beneficial effects of anti-IL-1β antibody treatment on these parameters, using again the adenine model of CKD (although with slight variations: earlier age, different background strain and 0.3% adenine diet for the first 10 days). Other studies have also demonstrated the salutary impact of suppressed inflammation on the anemic state in CKD, such as IL-6 deficient mice with CKD^24^. Also, pentoxifylline improves anemia and iron mobilization via suppression of IL-6 in CKD patients^44^. In this work, we show that administration of P2D7KK to CKD mice results in reduced systemic inflammatory response, as seen by decreased levels of peripheral blood WBC count, serum IL-6 and liver IL-1β, C-MYC, IL-6 and CRP (Fig. 5). The anti-inflammatory effect of P2D7KK was also observed in the kidney tissue: decreased levels of kidney TNF-α, IL-10, IL-1β and IL-6, and their downstream signaling molecules IRAK-4, MYD-88, c-MYC and p-STAT-3, were seen in the CKD-Ab in comparison to CKD group (Fig. 7). Consequently, kidney histology, as well as kidney TGF-β and macrophage markers levels showed decreased tubulointerstitial macrophage recruitment and attenuated interstitial fibrosis in the CKD-Ab group (Fig. 6). The improved kidney histology and inflammation were associated with a better kidney function, expressed as lower serum creatinine levels in CKD-Ab compared with CKD mice (Fig. 6B). Clinical data support the possible role of increased persistent low-grade inflammation through activation of the innate immune system (for example, in patients with rheumatoid arthritis) to the progression of CKD^45^. IL-1β activates a metabolic switch in stromal cells by upregulation of the MYC target gene, which promotes organoid hypertrophy, proximal tubule injury and fibrosis^23^. Moreover, selective depletion of the macrophage infiltrate in rats during the chronic phase of the disease, significantly decreased glomerular and interstitial fibrosis and improved renal function in association with a reduction in TGF-β1^46^.

The worsening anemia in RaKO mice with CKD and the mirror image of improved anemia with reduced inflammation when using an anti-IL-1β antibody indicate the possible protective effect of IL-1β inhibition on the anemic state in CKD (Fig. 8). However, no improvement in serum iron was observed in the CKD-Ab group, in spite of the reduction in liver hepcidin and ferritin and increased duodenal iron transporter (FPN-1) expression (Fig. 8D–G). This lack of increase in iron levels could be related to the limited iron content in the chow in our experiment (~ 75 ppm), in comparison to regular rodent chow used in laboratories (~ 200 ppm).

In the past years, new treatments (mostly prolyl hydroxylase inhibitors) have been developed to stabilize HIF-2α, which increases endogenous EPO production in patients with CKD induced anemia^47^. Here, anti-IL-1β administration to CKD mice also resulted in increased levels of kidney HIF-2α, leading to elevated serum EPO (Fig. 9A–C). Contrary to the decreased bone marrow EPOR and TFR levels in the CKD group, CKD-Ab group showed upregulated levels of these receptors (Fig. 9D,E). Hence, anti-IL-1β treatment not only increased EPO levels but may have ameliorated bone marrow resistance by increasing EPOR expression. Moreover, the upregulation of bone marrow TFR may facilitate iron uptake and utilization for red blood cells production.

Clinical studies using anti-IL1 therapy to prevent adverse cardiovascular outcomes have been published over the past years^21^. A secondary analysis of the CANTOS trial, which concentrated on the subgroup of patients with mild CKD, showed again a positive effect on the prevention of adverse cardiovascular outcomes in this subpopulation, but did not identify beneficial (or adverse) effect on renal outcomes^48^. However, the study was not powered or designed to examine this question.

In summary, this study describes the distinct role of IL-1 in CKD-associated progressive renal damage and anemia. Exaggerated inflammation was associated with a higher degree of renal insufficiency and anemia. In addition to the well-known hepcidin mediated decreased iron mobilization, the normal response to anemia induced hypoxia is deranged in CKD and even more accentuated when inflammation is increased in RaKO-CKD: renal HIF-2α and EPO, BM EPO-R and TFR are not upregulated, which further exacerbate anemic tendency. Complementary to these findings, administration of P2D7KK, a novel anti-IL1$\upbeta$ monoclonal antibody to CKD mice results in enhanced serum EPO synthesis and availability, via increased renal HIF-2α synthesis and attenuated inflammatory suppression, thus restoring EPOR and TFR bone marrow signaling, as well as suppressed hepcidin levels, thereby improving CKD related anemia. By doing so, P2D7KK treatment may lead to a better response to exogenous EPO and iron supplementation. Therefore, novel treatments to reduce inflammation, such as P2D7KK or other compounds may potentially be an alternative and/or adjunctive therapy for patients with CKD suffering from anemia.

## Methods

### Animals

This study was approved by the Ben-Gurion University of the Negev Animal Use and Care Committee protocol number IL-39–07-2018. All protocols comply with the NIH Guidelines. Animals were housed in standard laboratory cages. Food and water were given ad libitum. The study was carried out in compliance with the ARRIVE (Animal Research: Reporting of In Vivo Experiments) guidelines (https://arriveguidelines.org/arrive-guidelines).

In the first set of experiments, 12 weeks old male BALB/c WT mice (Harlan Laboratories Inc. Rehovot, Israel) and IL-1 receptor-antagonist knockout (IL-1Ra-KO) mice (BALB/c background) were divided into 4 groups (n = 6–10 in each group): WT (WT mice, control diet), WT-CKD (WT mice, adenine diet), RaKO (IL-1Ra-KO mice, control diet), RaKO-CKD (IL-1Ra-KO mice, adenine diet). CKD was induced by adenine diet^49^ (0.2% adenine, 0.9% phosphorus, 75 ppm iron) while control groups were fed with a control diet (0.3% phosphorus, 75 ppm iron). Mice were sacrificed using anesthesia with ketamine and xylazine after 10 weeks, collecting: blood, liver, kidney and bone marrow aspirate.

In the second series of experiments, 7–8 weeks old male C57BL/6 mice (Harlan Laboratories Inc. Rehovot, Israel) were divided into 3 groups (n = 6–10 in each group): C, CKD and CKD-Ab. CKD was induced by a 0.3% adenine diet, which also contained 0.9% phosphorus and 75 ppm iron given for 10 days and then a 0.2% adenine diet and unchanged phosphorus and iron, given for additional 6 days. Control groups were fed with a control diet (0.3% phosphorus, ~ 75 ppm iron). All diets were purchased from Envigo Teklad, (Huntingdon, UK). A dose of 5 mg/kg of anti-IL-1β antibody P2D7KK was given i.p. to CKD-Ab mice twice a week, while C and CKD groups were injected i.p with saline. P2D7KK dose was determined according to the previous study and advice of Goh et al. ^20^. Mice were sacrificed after 16 days, collecting: blood, liver, duodenum, kidney and bone marrow aspirate. The experiments are summarized in Supplemental Table 1.

### Blood analyses

Complete blood count was assessed from tail bleed collected into EDTA-coated capillary tubes (Pro-Vet laboratory, Yodfat, Israel, GENESIS analysis system, Oxford Science, Oxford, USA). Kidney functions and serum iron levels from were analyzed using the AU2700 analyzer (Beckman-Coulter, CA, USA). Serum erythropoietin (EPO) levels were determined by enzyme-linked immunosorbent assays (Quantikine Mouse Epo kit, R&D Systems, Minneapolis, MN. Sensitivity: 47.0 pg/mL; intra- and inter-assay coefficients of variation: < 4.4% and 9.7%, respectively). Serum IL-6 concentrations were performed by mouse IL-6 ELISA Max Set Deluxe Kits (Biolegend, San Diego, CA, USA. Sensitivity: 2 pg/mL).

### RNA extraction and real-time PCR

Assays were performed with power SYBR green PCR master mix (Applied Biosystems, Foster City, CA, USA) as previously described^50^ using the ABI Prism 7300 Sequence Detection System (Applied Biosystems, Foster City, CA, USA). Primers for quantification of HIF-2α, CRP, Hepcidin, IL-6, EPOR, TFR, c-MYC, IL-1β, FPN-1, IRAK-4, MYD-88, TGF-β, CD-163, CD-11c, F4/80, Ferritin, TNF-α, IL-10 and β-actin (Sigma-Aldrich, Rehovot, Israel) are summarized in Supplemental Table 2.

### Western immunoblot analysis

The following antibodies were used for evaluation of the kidney extracts: STAT3, p-STAT3 (polyclonal, Cell Signaling Technology Inc. Danvers, MA), HIF-2α (polyclonal, Novus Biologicals, Littleton, CO) and β-actin (Clone C4, MP Biomedical Solon, OH, USA), as previously described^51^.

### Kidney histology

Kidney segments were fixed in 4% formalin for 48 h, then embedded in paraffin and cut. Kidney sections were deparaffinized, rehydrated and stained with hematoxylin & eosin (Sigma-Aldrich, Saint Louis, MO, USA) or Masson’s trichrome (Bio-Optica, Milano, Italy). Fibrotic area quantification of Masson’s trichrome staining was performed as described by Chen et al. using an *ImageJ* software^52^.

Immunohistochemistry staining was performed as previously described ^50^, primary antibodies against F4/80 (BM-8) (diluted 1:30; Santa Cruz biotechnology Inc., Dallas Texas, USA) and Myeloperoxidase (MPO) (diluted 1:50; Abcam, Cambridge, UK) were used for the immunohistochemistry staining. For image processing, *Cellsense Entry* software (MATIMOP, Tel Aviv, Israel) was used.

### Data analysis

One-way ANOVA with post hoc Tukey's test was used to determine significant differences based on multiple comparisons. The null hypotheses were rejected at the 5% level. Values along the manuscript are presented as means ± standard errors. Different letters above bars indicate a significant difference between groups and same letters indicate no significant difference.

## Supplementary Information

Supplementary Information
