# Supplementary material for: Experimental modulation of Interleukin 1 shows its key role in chronic kidney disease progression and anemia
Source: Sci Rep. 2021 Mar 18;11:6288. doi: 10.1038/s41598-021-85778-2 (PMC7973507; doi:10.1038/s41598-021-85778-2)

Supplementary Materials

Experimental modulation of Interleukin 1 shows its key role in chronic kidney disease progression and anemia.

Inbar Bandach, Yael Segev, Daniel Landau

Table of Contents

Methods **2-4**

Supplemental Table 1. Summary of experiments 2

Supplemental Table 2. Primers used for real-time PCR analysis 3-4

Results **5-12**

Supplementary Figure S1. Summary of experiments and main results 5

Supplementary Figure S2. Inflammation phenotype 6

Supplementary Figure S3. Adenine crystal 7

Supplementary Figure S4. Body weight gain (g) of mice on regular (C) or adenine diet, injected with saline (CKD) or an anti-IL1 monoclonal antibody (CKD-Ab)........ 8

Supplementary Figure S5. Full-length original western blots for STAT 3 and p-STAT 3 referred to in Figure 2I........ 9

Supplementary Figure S6. Full-length original western blots for HIF-2α referred to in Figure 4B........ 10

Supplementary Figure S7. Full-length original western blots for STAT 3 and p-STAT 3 referred to in Figure 7H........ 11

Supplementary Figure S8. Full-length original western blots for HIF-2α referred to in Figure 9B........ 12

Supplemental Table 1. Summary of experiments

Exp. No.	Group	Animals	Diet	Treatment	Sample size	Figure numbers
#1	WT	BALB/c WT mice	Control	-	n=6-10/ group	Figures 1-3 and Supplementary Figure S1
	WT-CKD	BALB/c WT mice	Adenine	-		
	RaKO	IL-1 receptor-antagonist knockout mice on BALB/c background	Control	-		
	RaKO-CKD	IL-1 receptor-antagonist knockout mice on BALB/c background	Adenine	-		
#2	C	C57BL/6 mice	Control	Saline	n=6-10/ group	Figures 4-8 and Supplementary Figure S2
	CKD	C57BL/6 mice	Adenine	Saline		
	CKD-Ab	C57BL/6 mice	Adenine	anti-IL-1β antibody (P2D7KK)		

Supplemental Table 2. Primers used for real-time PCR analysis

Gene	SENSE	ANTI SENSE
Hif-2α	AGTAGCCTCTGTGGCTCCAA	TCCAGGGCATGGTAGAACTC
CRP	CCATTTCTACACTGCTCTGAGCAC	CCAAAATATGAGAATGTCGTTAGAGTTC
Hepcidin	CTGAGCAGCACCACCTATCTC	TGGCTCTAGGCTATGTTTTGC
IL-6	CTATACCACTTCACAAGTCGG AGG	TGCACAACTCTTTTCTCATTTCC
EPOR	CAACAGCGGACACATCGAGTT	TGCGGTGATAGCGAGGAGA
TFR	CATGAGGGAAATCAATGATCG TA	GCCCCAGAAGATATGTCGGAA
c-MYC	CGGACACACAACGTCTTGGAA	AGGATGTAGGCGGTGGCTTTT
IL1-β	ACAACCACGGCCTTCCCTACTT	CACGATTTCCCAGAGAACATGTG
FPN-1	TCGGTTCCTCTCACTCCT GT	GTGGAGAGAGAGTGGCCA AG
IRAK-4	GTCATGACCAGCCGAATCGTG	CAGACACTGGTCAGCAGCAGA
MYD-88	CGATTATCTACAGAGCAAGGAATG	ATAGTGATGAACCGCAGGATAC
TGF-β	GCAACATGTGGAACTCTACCAGA	GACGTCAAAAGACAGCCACTCA
CD-163	GGGTCATTCAGAGGCACACTG	CTGGCTGTCCTGTCAAGGCT
CD-11c	CTGGATAGCCTTTCTTCTGCTG	GCACACTGTGTCCGAACTC

F4/80	CGTCAGGTACGGGATGAATATAAG	CTATGCCATCCACTTCCAAGAT
Ferritin	GATCAACCTGGAGTTGTATGCC	CTCCCAGTCATCACGGTCTG
TNF-α	GAATGGGTGTTCATCCATTCT	ACATTCGAGGCTCCAGTGAATTCG
IL-10	GTTACTTGGGTTGCCAAG	TTGATCATCATGTATGCTTC
β-Actin	GGTCTCAAACATGATCTGGG	GGGTCAGAAGAATTCCTATG

Supplementary Figure S1. Summary of experiments and main results.

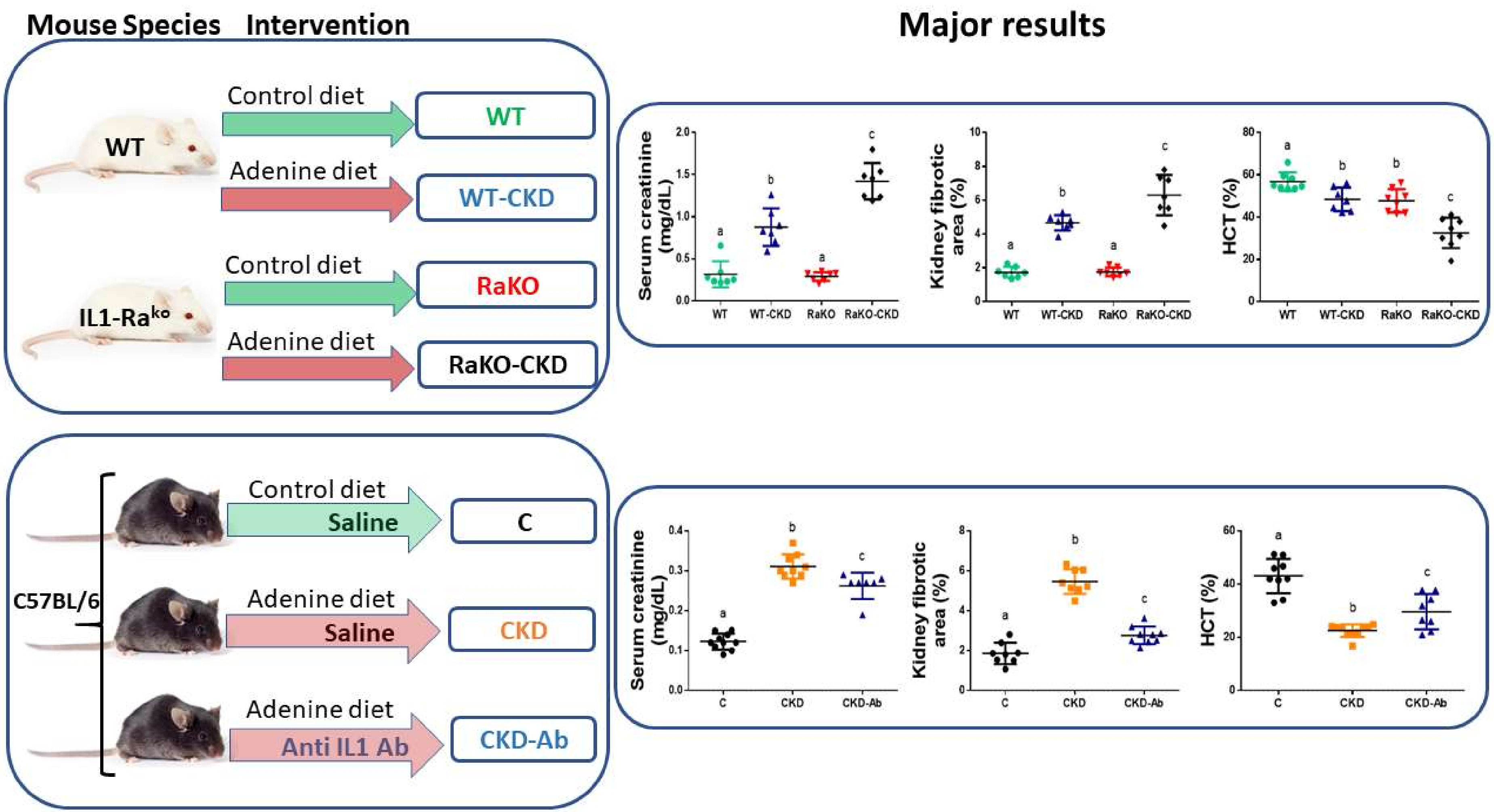

Supplementary Figure S2. Inflammation phenotype. Ankle joints of wild type (WT), WT-CKD, RaKO and RaKO-CKD mouse.

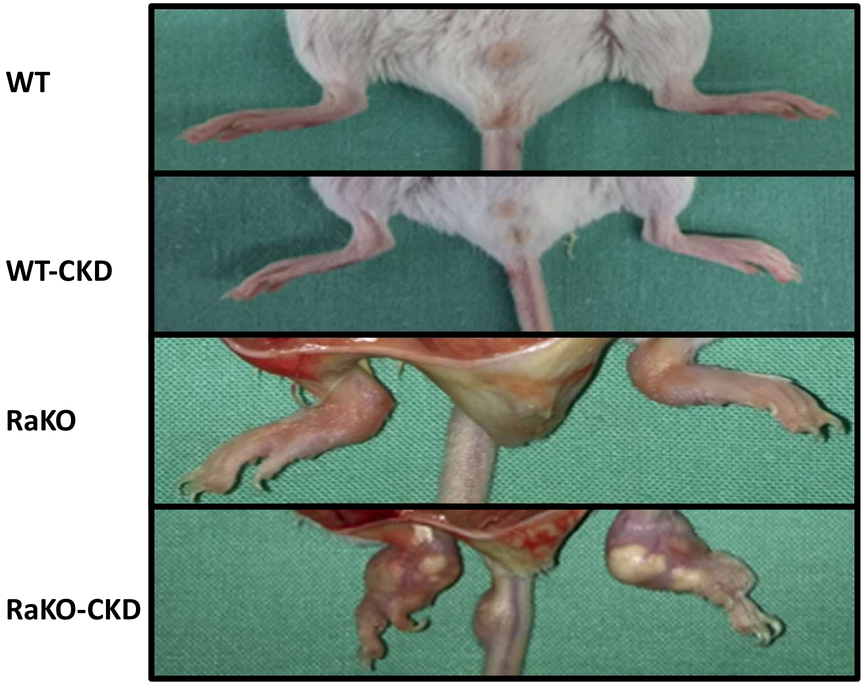

Supplementary Figure S3. Adenine crystal (arrowhead) in hematoxylin & eosin stained histological kidney section from WT-CKD animal.

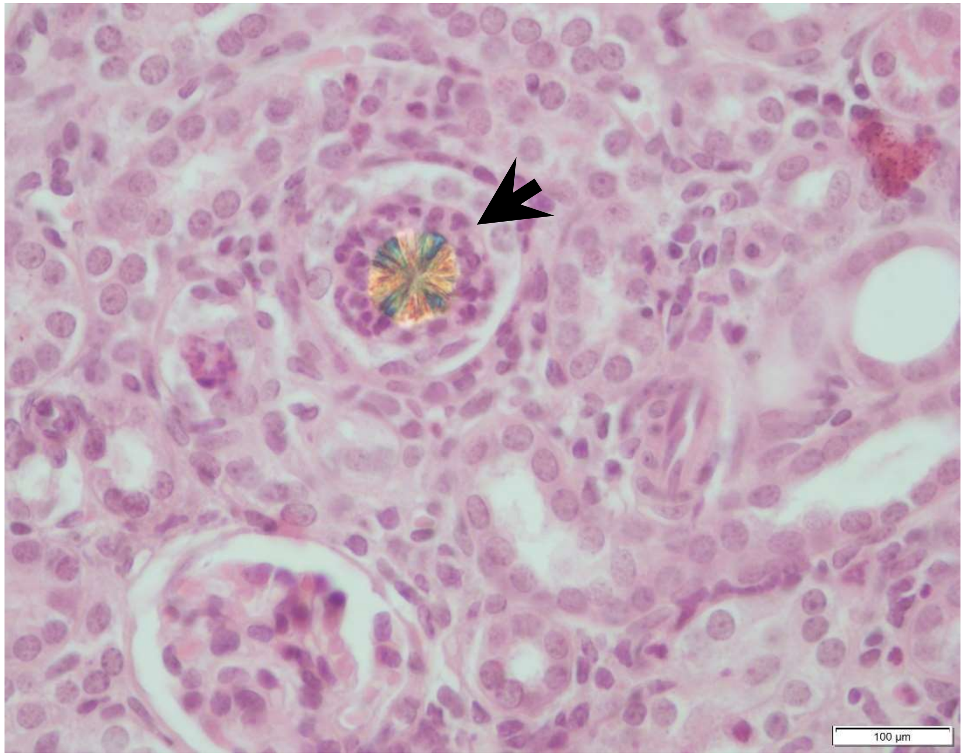

Supplementary Figure S4. Body weight gain (g) of mice on regular (C) or adenine diet, injected with saline (CKD) or an anti-IL1 monoclonal antibody (CKD-Ab).

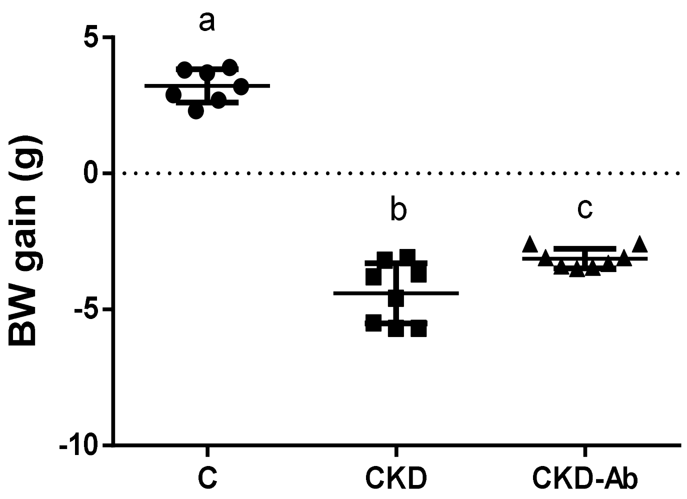

Supplementary Figure S5. Full-length original western blots for STAT 3 and p-STAT 3 referred to in Figure 2I. After the size marker, columns from left to right belong to: WT (1-2), WT-CKD (3-4), RaKO (5-6) and RaKO-CKD (7-8):

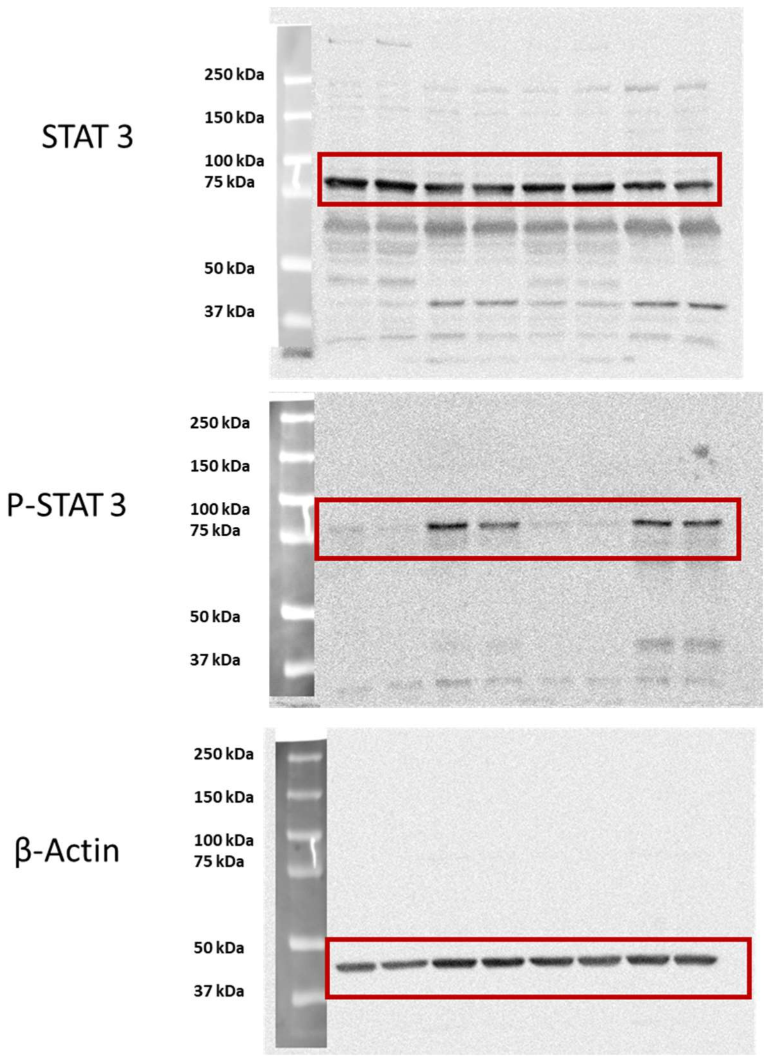

Supplementary Figure S6. Full-length original western blots for HIF-2α referred to in Figure 4B. After the size marker, columns from left to right belong to: WT (1-2), WT-CKD (3-4), RaKO (5-6) and RaKO-CKD (7-8):

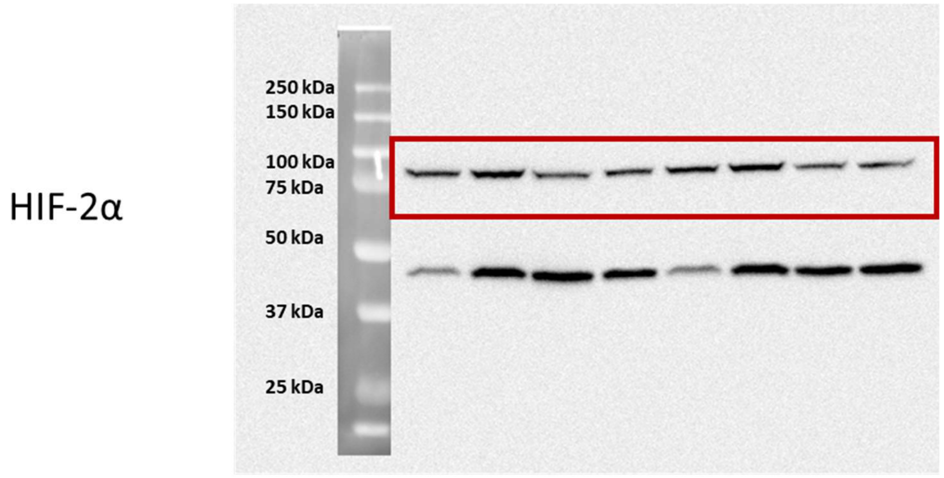

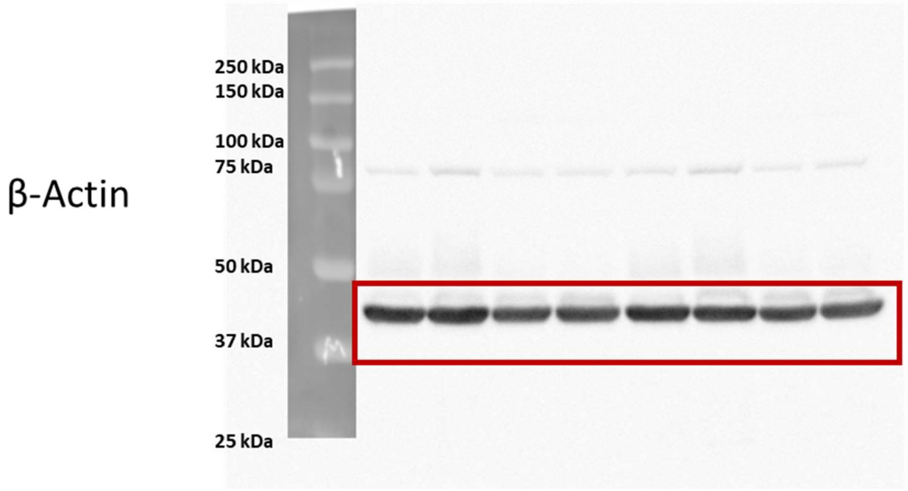

Supplementary Figure S7. Full-length original western blots for STAT 3 and p-STAT 3 and β actin, referred to in Figure 7H. After the size marker, columns from left to right belong to: C (1-3), CKD (4-6), and CKD-Ab (7-9):

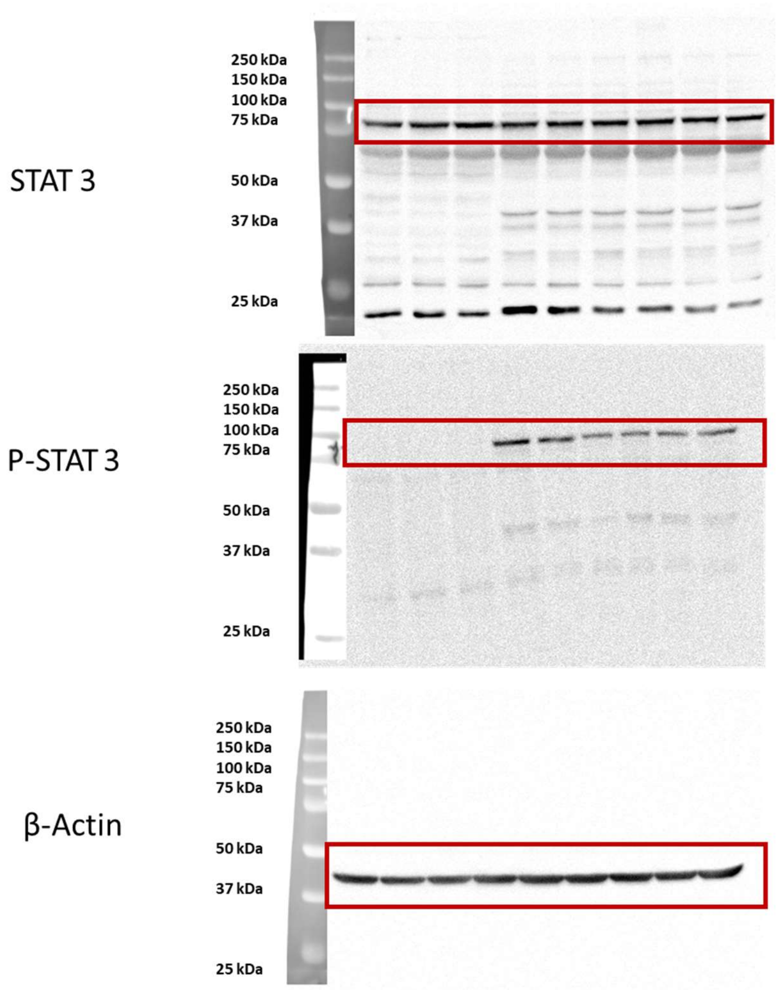

Supplementary Figure S8. Full-length original western blots for HIF-2α referred to in Figure 9B. After the size marker, columns from left to right belong to: C (1-3), CKD (4-6), and CKD-Ab (7-9):

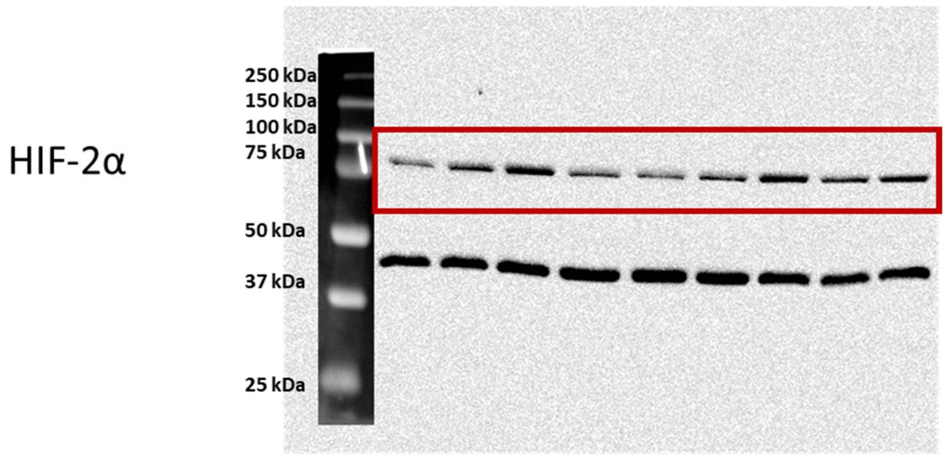

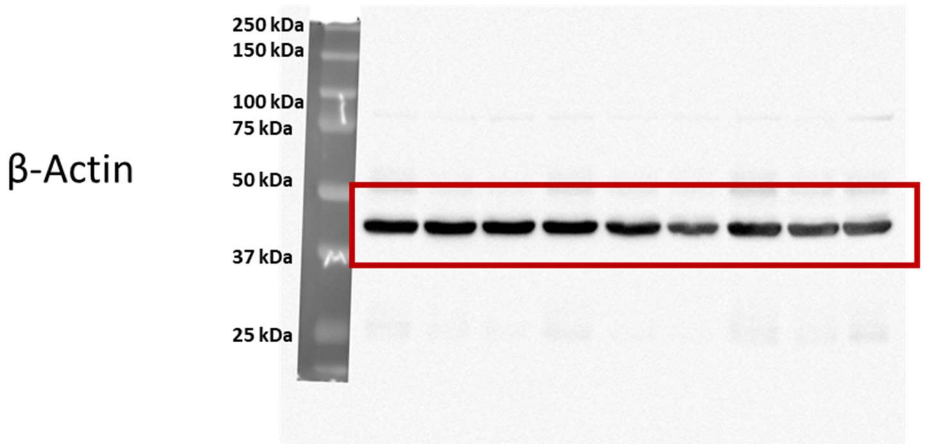

Supplement: Supplementary file 1 — Supplementary Information [file 41598_2021_85778_MOESM1_ESM.pdf]
